# Publication activity of chief and consultant general/visceral surgeons in German university hospitals—a ten-year analysis

**DOI:** 10.1007/s00423-021-02241-6

**Published:** 2021-07-26

**Authors:** Eva C. Böckmann, E. S. Debus, R. T. Grundmann

**Affiliations:** Department of Vascular Medicine, University Heart Center (UHC), University Hospital Hamburg-Eppendorf, Hamburg, Germany

**Keywords:** Abdominal surgery, General surgery, Publication, Impact factor, University

## Abstract

**Purpose:**

The publication activity of 38 German general/visceral surgery university departments, documented by first or last authorship from staff surgeons (chief and consultants), was evaluated.

**Methods:**

The observation period extended from 2007 to 2017 and all PubMed-listed publications were considered. Impact factor (IF) was evaluated through the publishing journal’s 5-year IF in 2016, as was the IF for each individual publication. Ranking was expressed in quartiles.

**Results:**

The staff surgeons of the 38 departments comprised 442 surgeons, of which only 351 (79.4%) were active as first or last authors. Four thousand six hundred and ninety-nine publications published in 702 journals were recorded. The four leading departments in publication number published as much as the last 20 departments (1330 vs. 1336 publications, respectively). The mean of the first (most active) department quartile was 19.6 publications, the second 15.4, the third 11.0, and the last quartile 7.6 per publishing surgeon. The total cumulative impact factor was 14,130. When examining the mean number of publications per publishing surgeons per the 10 year period, the mean of the first quartile was 57.9 cumulative IF, the second 45.0, the third 29.5, and the fourth quartile 17.1. With 352 (7.5%) publications, the most frequently used journal was *Chirurg*, followed by *Langenbeck’s Archives of Surgery* with 274 (5.8%) publications. Pancreas-related topics led in terms of publication number and IF generated per individual publication.

**Conclusion:**

A significant difference in publication performance of individual departments was apparent that cannot be explained by staff number. This indicates that there are as yet unknown factors responsible for minor publication activity in many university departments.

Reputation and funding of university hospital departments increasingly depends on publication performance. Considerable difference in the publication performance of individual departments has been demonstrated by Putzer et al. [1] in the field of anesthesiology with a non-anonymized ranking of 45 university hospital departments in Germany, Austria, and Switzerland between 2001 and 2010. No conclusions were drawn, perhaps because contentious discourse about causal factors could have been sharpened, but also because the study did not take hospital structure or employee number into account. Benchmarking, as carried out by Putzer et al., has not yet been done regarding publication activity in German general and visceral surgery university departments. However, other surgical fields in German universities have undergone analysis in terms of publication in the past decade. These include plastic surgery by Schubert et al. [2], vascular surgery by Debus et al. [3] as well as by Haffke et al. [4] and academic heart surgery [5]. Large discrepancies between the individual departments in all of these fields were also reported, whereby differences were mainly attributed to structure. Independent departments showed a significantly higher publication activity than subordinate departments or sections.

The present study was designed to investigate the publication activity in German university visceral surgery. To enable future comparisons of the different surgical disciplines, the methodology of Schubert et al. [2], Haffke et al. [4], and Debus et al. [3, 5] was applied, which exclusively focuses on staff surgeons consisting of chief and consultants. The intent was to investigate whether, as suspected by Putzer et al. [1], publication performance primarily depends on the size of the department, or to whether performance relies on preferentially used journals for publishing, or if the publication topic is a deciding factor in performance.

## Materials and methods

The publication performance of staff surgeons consisting of the chief and consultants of all 38 German general and visceral surgery university departments was recorded. Staffing was drawn from department websites and the key date for all consideration (staffing and publications) was July 1, 2017, with the observation period spanning 10 years from January 1, 2007. Only staff members listed on the department’s website on July 1st were considered as first or last authors. Staff members who had previously left the department were not considered, so that a snapshot on July 1, 2017 was taken. The total number of publications during the entire period 2007 through 2017 was compiled. Publications were recorded when listed in PubMed (Medline) in the form of original papers and reviews with an abstract. There were no exclusion criteria with regard to the choice of topics by the authors. Comments and answers to inquiries were excluded. For each member of the staff surgeons claiming first or last authorship, all publications fulfilling these requirements were recorded (journal, publication title, month, and year of publication). Duplicate attribution for individual publications from the same department, where first and last authorship were claimed by staff surgeons, was factored out in calculating the department’s total publication number, the total impact factor, publishing journal, and publication focus. If an author changed jobs during the 10-year period, the author's publication output was assigned to the department where the author was employed as of July 1, 2017. In a second step, the journal’s 5-year impact factor (IF) for 2016 was recorded via the “Web of Science” under “Journal Citation Reports,” “Journals by Rank,” or “Select Journals.” Journals unlisted in the “Journal Citation Reports” database or for which a 5-year impact factor for 2016 could not be determined were not considered, nor were Eigenfactor scores taken into consideration.

Due to the multitude of surgical fields, a compromise was necessary to determine publication focus. The five most common focal points were:

- Hepatobiliary
- Colorectal (appendix, chronic inflammatory bowel disease, ileum, colon, rectum)
- Upper gastrointestinal tract (esophagus, stomach, duodenum)
- Pancreas
- Transplantation (general transplant research, liver, kidney, pancreas transplantation)

All other fields (including endocrine surgery) were grouped under the heading “other”.

### Ranking

The departments were ranked from 1 to 38 according to publication number (total and average per member), total acquired IF, and IF of individual publications. Quartiles were created for each ranked parameter, whereby quartiles 1 and 2 each consisted of 9 departments and quartiles 3 and 4 consisted of 10 departments each. Quartile 1 reflected the greatest, quartile 4 the least publication activity. Since the departments were not uniformly ranked for all four parameters, the quartile in which a department appears is parameter dependent.

### Statistics

Range and interquartile range (IQR) are expressed. Groups were checked for significant differences using Student’s *t* test, *P* < 0.05 being chosen as the level of significance. Correlation between parameters was calculated using the Pearson correlation coefficient (*r*). Values < 0.3 were interpreted as a weak relationship, between 0.3 and 0.6 as a moderate relationship and > 0.6 as a close relationship, assuming a significance of *P* < 0.05. The quartiles were examined for differences using the Kruskal–Wallis test and Mann–Whitney *U* test.

## Results

### Total publications

**Fig. 1:**
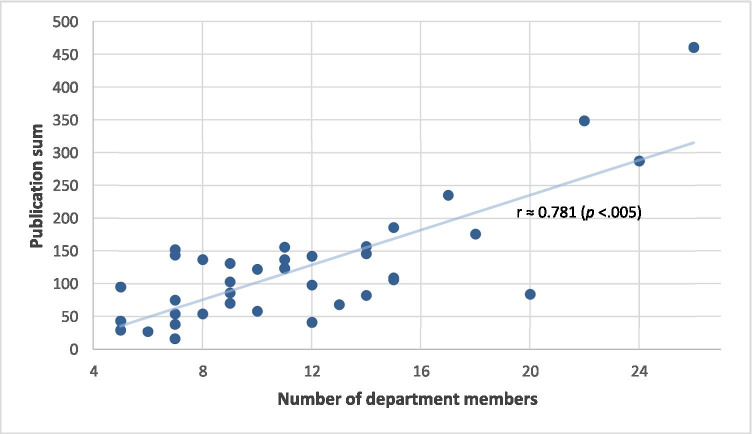
Relationship between sum of publications in individual departments and number of department members

**Fig. 2:**
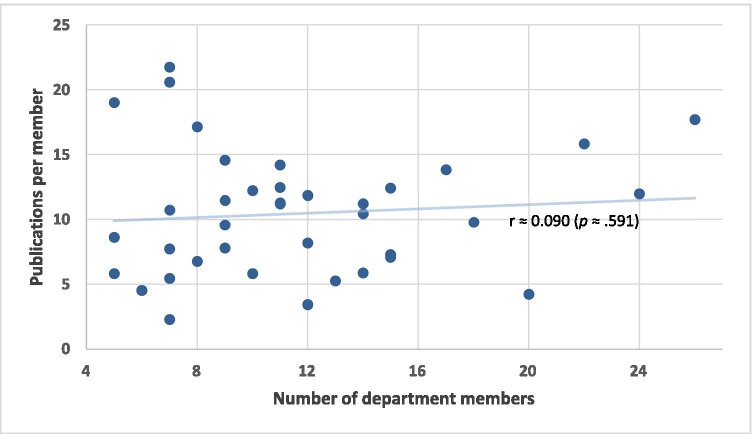
Relationship between number of department members and publication number per member

The publication activity of the individual departments is shown in Table 1. The staff surgeons (chief and consultants) of the 38 departments totaled 442 members, averaging 11.6 members per department (range 5–26; IQR 6.8). Only 351 (79.4%; range 28.6–100%) members were active as first or last author. A total of 4699 publications were recorded, averaging 123.7 publications per department, ranging from 460 to 16 publications per department, with a median of 108 (IQR 77). The leading 4 departments in overall publication published as much as the last 20 departments together (1330 and 1336 publications, respectively). The number of publications in a department depended on the member number (Fig. 1), but there was no relationship between the number of members and the number of publications per member (Fig. 2). The same non-significance was apparent for the number of members and the proportion of members with publication activity, with a correlation coefficient of 0.113 (*P* ≈ 0.50). Larger and smaller departments did not differ significantly in the percentage of their publication-active members.

**Table 1:** Publication activity in the 38 German university departments of general/visceral surgery

University	Number of department members (proportion of members with publication activity)	Publication sum	Cumulative IF	Ø Publications per member	Ø Publications per publishing member	Cumulative IF per member	Cumulative IF per publishing member
Aachen	8 (100%)	137	349.3	17.1	17.1	43.7	43.7
Berlin CBF	5 (100%)	95	236.9	19.0	19.0	47.4	47.4
Berlin CCM + CVK	24 (100%)	287	768.5	12.0	12.0	32.0	32.0
Bochum	20 (50%)	84	160.9	4.2	8.4	8.0	16.1
Bonn	9 (100%)	86	262.5	9.6	9.6	29.2	29.2
Dresden	15 (73%)	186	651.5	12.4	16.9	43.4	59.2
Düsseldorf	12 (83%)	98	353.9	8.2	9.8	29.5	35.4
Erlangen	15 (80%)	106	286.3	7.1	8.8	19.1	23.9
Essen	14 (57%)	157	347.4	11.2	19.6	24.8	43.4
Frankfurt	7 (71%)	54	99.5	7.7	10.8	14.2	19.9
Freiburg	14 (85%)	146	543.7	10.4	12.2	38.8	45.3
Gießen	7 (42%)	16	34.5	2.3	5.3	4.9	11.5
Göttingen	15 (60%)	109	470.2	7.3	12.1	31.3	52.2
Greifswald	8 (75%)	54	106.8	6.8	9.0	13.4	17.8
Halle	5 (40%)	29	46.1	5.8	14.5	9.2	23.1
Hamburg	9 (88%)	131	489.4	14.6	16.4	54.4	61.2
Hannover	14 (78%)	82	211.5	5.9	7.5	15.1	19.2
Heidelberg	26 (84%)	460	1905.7	17.7	20.9	73.3	86.6
Homburg	7 (28%)	38	79.0	5.4	19.0	11.3	39.5
Jena	11 (81%)	137	284.2	12.5	15.2	25.8	31.6
Kiel	10 (90%)	58	177.6	5.8	6.4	17.8	19.7
Köln	12 (100%)	142	410.3	11.8	11.8	34.2	34.2
Leipzig	9 (66%)	103	304.8	11.4	17.2	33.9	50.8
Lübeck	11 (63%)	124	337.8	11.3	17.7	30.7	48.3
Magdeburg	7 (85%)	144	236.4	20.6	24.0	33.8	39.4
Mainz	7 (100%)	152	336.5	21.7	21.7	48.1	48.1
Mannheim	11 (100%)	156	553.9	14.2	14.2	50.4	50.4
Marburg	7 (71%)	75	256.3	10.7	15.0	36.6	51.3
München LMU	17 (88%)	235	795.0	13.8	15.7	46.8	53.0
München TU	22 (100%)	348	1238.4	15.8	15.8	56.3	56.3
Münster	9 (100%)	70	239.5	7.8	7.8	26.6	26.6
Oldenburg	6 (66%)	27	35.3	4.5	6.8	5.9	8.8
Regensburg	10 (80%)	122	403.5	12.2	15.3	40.4	50.4
Rostock	5 (100%)	43	90.6	8.6	8.6	18.1	18.1
Tübingen	18 (83%)	176	514.8	9.8	11.7	28.6	34.3
Ulm	13 (61%)	68	136.5	5.2	8.5	10.5	17.1
Witten/Herdecke	12 (41%)	41	125.3	3.4	8.2	10.4	25.1
Würzburg	11 (100%)	123	249.7	11.2	11.2	22.7	22.7

### Department benchmarking—publication number per publishing member

**Fig. 3:**
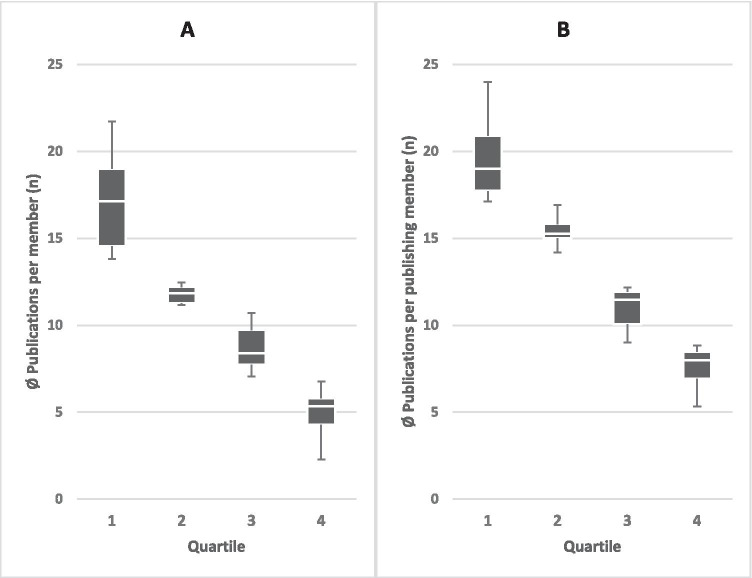
Benchmarking—**A** number of publications per department member compared to **B** number of publications per publishing member in the department. Quartiles 1 and 2 include 9 departments, quartiles 3 and 4 include 10 departments. Boxplots show the middle 50%, divided by the median (white line), whiskers show minimum and maximum of each quartile

According to publication activity, departments were assigned to appropriate quartiles, with quartile 1 as most and quartile 4 as least active. To avoid a bias in benchmarking due to the number of members in the department, the number of publications was calculated per publishing member. Considerable differences between the university departments were apparent (Fig. 3A and B). The mean number of publications per member in the first quartile was 17.2 (range 21.7–13.8; median 17.1; IQR 4.4), the second 11.8 (range 12.5–11.2; median 11.8; IQR 0.9), the third 8.7 (range 10.7–7.1; median 8.4; IQR 2.0), and the last quartile was 4.9 (range 6.8–2.3; median 5.3; IQR 1.5) (Fig. 3A). The leading 9 departments in the first quartile published approximately 3 times more per member, compared to the last 10 departments in the fourth quartile. Adjusted to the number of publishing members, the quartile differences were slightly smaller, but still clear (Fig. 3B). Here, the mean of the first most active quartile was 19.6 (range 24.0–17.1; median 19.0; IQR 3.2) publications, the second 15.4 (range 16.9–14.2; median 15.3; IQR 0.8), the third 11.0 (range 12.2–9.0; median 11.5; IQR 1.9), and the last with 7.6 (range 8.8–5.3; median 8.0; IQR 1.5) publications per publishing member. The IQR of the average publications per member of all the departments was 5.6 vs. 7.9 for the publications per publishing member.

### Total impact factor

The total (cumulative) impact factor for a total of 4699 publications was 14,130. The generated IF per department ranged from 1906 to 35. The mean cumulative IF per department was 372 (median 285; IQR 290). The IF per member ranged from 73.3 to 4.9; the mean was 32.0 (median 29.3) per member. As apparent for the sum of publications, the sum of the cumulative impact factor for a department depended on the number of members (correlation coefficient *r* = 0.762; *P* < 0.05). A moderate correlation between the number of department members and IF per publication was also apparent (*r* = 0.451; *P* < 0.005).

### Department benchmarking—cumulative impact factor per member

**Fig. 4:**
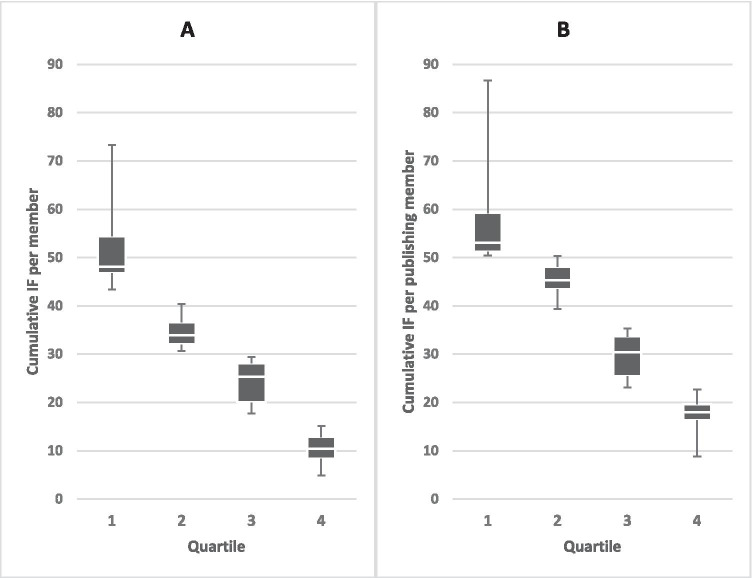
Benchmarking—**A** cumulative impact factor (IF) per department member compared to **B** cumulative IF per publishing member in the department. Quartiles 1 and 2 include 9 departments, quartiles 3 and 4 include 10 departments. Boxplots show the middle 50%, divided by the median (white line), whiskers show minimum and maximum of each quartile

The mean of the first quartile (most active) for the cumulative IF per member, was 51.5 (range 73.3–43.4; median 48.1; IQR 7.6), the second was 34.6 (range 40.4–30.7; median 33.9; IQR 4.6), the third was 24.2 (range 29.5–17.8; median 25.3; IQR 8.1), and the last was 10.3 (range 15.1–4.9; median 10.5; IQR 4.5) (Fig. 4A). The leading 9 departments (1st quartile) generated about 5 times the IF per member compared to the last 10 departments (4th quartile). Adjusted to the number of publishing members, the quartile differences were smaller, but still clear (Fig. 4B). The mean of the first quartile (most active) was 57.9 (range 86.6–8.0; median 53.0; IQR 8.0), the second was 45.0 (median 45.3; IQR 4.6), the third was 29.5 (range 35.4–23.1; median 30.4; IQR 8.2), and the fourth quartile was 17.1 (median 18.0; IQR 3.3) per publishing member. The cumulative IF per publishing member was about 3 times more in the leading 9 departments (1st quartile), compared to the last 10 departments (4th quartile). The IQR of the average cumulated IF per member for all the departments was 24.2 vs. 27.0 for the IF per publishing member.

### Department benchmarking—impact factor per publication

**Fig. 5:**
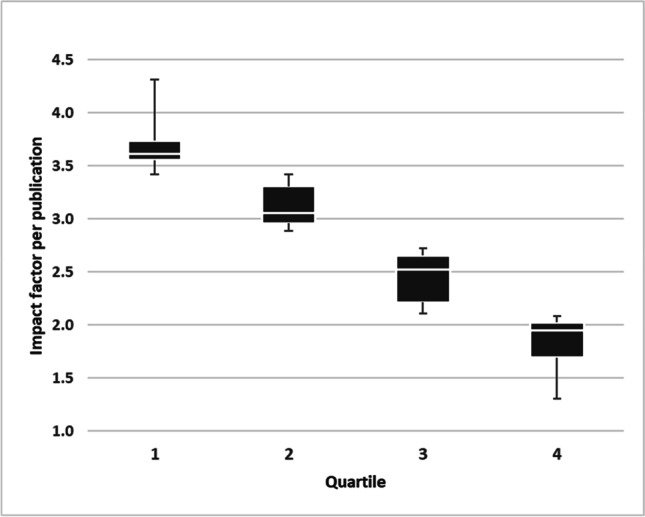
Benchmarking – mean impact factor per publication. Quartiles 1 and 2 include 9 departments, quartiles 3 and 4 include 10 departments. Boxplots show the middle 50%, divided by the median (white line), whiskers show minimum and maximum of each quartile

The IF span for single publications achieved by a department ranged on average from 4.3 to 1.3. The mean IF per publication was 3.0, the median 2.7. The range of the first quartile was from 4.3 to 3.4 with a mean of 3.7 (Fig. 5).The second quartile covered the range from 3.4 to 2.9 with a mean of 3.1, the mean IF per publication in the third quartile ranged from 2.7 to 2.1 with a mean IF per publication of 2.4, the last quartile ranged from 2.1 to 1.3 with an average of 1.8.

### Ranking of journals according to number of published articles and generated cumulative impact factor

The 4699 publications appeared in a total of 702 PubMed-listed journals. No more than 10 publications appeared in 630 (89.7%) journals. In 312 journals (44.4%), only one publication appeared. The 20 most frequently used journals are listed in Table 2, together with their impact factors and cumulative IF (C-IF) generated by the publications. Two thousand and sixty-six (44.0%) publications appeared in these 20 journals. With 352 (7.5%) publications, *Chirurg* published most frequently with a C-IF of 210.This was followed by *Langenbeck’s Archives of Surgery* with 274 (5.8%) publications and a C-IF of 634.9 and the *Zentralblatt für Chirurgie* with 234 (4.98%) publications and a C-IF of 141. Further information regarding publication frequency is shown in Table 2.

**Table 2:** Twenty most frequently used journals, with their impact factors (IF) and the cumulatively generated impact factor (C-IF)

Rank	Number (%) of publications	Journal	5-year-IF 2016	Cumulative IF (C-IF)	Rank acc. to C-IF
1	352 (7.49%)	Chirurg	0.60	210	14
2	274 (5.83%)	Langenbeck ‘s Arch Surg	2.32	635	2
3	234 (4.98%)	Zentralbl Chir	0.60	141	22
4	109 (2.32%)	Int J Colorectal Dis	2.58	281	7
5	98 (2.09%)	Ann Surg	9.41	922	1
6	91 (1.94%)	Transplant Proc	1.02	93	35
7	89 (1.89%)	J Gastrointest Surg	2.99	266	9
8	84 (1.79%)	Surgery	4.16	349	4
9	83 (1.77%)	World J Surg	2.92	243	10
10	74 (1.57%)	Surg Endosc	3.74	277	8
11	72 (1.53%)	Ann Surg Oncol	4.39	316	6
12	71 (1.51%)	Transpl Int	2.93	208	15
13	62 (1.32%)	Transplantation	3.73	231	12
14	60 (1.28%)	PLoS One	3.39	204	16
15	58 (1.23%)	Br J Surg	6.08	353	3
16	56 (1.19%)	J Surg Res	2.21	124	27
17	54 (1.15%)	BMC Cancer	3.65	197	17
18	49 (1.04%)	Pancreas	3.30	162	20
18	49 (1.04%)	Eur Surg Res	1.64	80	39
20	47 (1.00%)	World J Gastroenterol	3.18	149	21

*Annals of Surgery* took first place in terms of generated IF, with a C-IF of 922 (6.5% of the total IF), even though it only ranked fifth according to the number of publications. In terms of both frequency and C-IF, *Langenbeck’s Archives of Surgery* placed second with a C-IF of 635 (4.5% of the total IF). The *British Journal of Surgery* was third, with a C-IF of 353 (2.5% of the total IF), and 15th in number of publications (Table 2).

### Focus of publications

The percentage of publications dedicated to specific surgical fields is shown in Table 3. Pancreas was the subject of approximately 20% of all publications, while only 9.4% of all publication was devoted to the upper gastrointestinal tract. Pancreas also led in cumulative IF and came in first place with an average IF of 4.0 per individual publication.

**Table 3:** Ranking according to number of publications in different surgical fields with cumulative IF and mean IF of individual publications (*IF,* impact factor)

Field	Number of publications	Cumulative IF	Mean IF of individual publications
Pancreas	905 (19.3%)	3635 (25.7%)	4.0
Transplantation	733 (15.6%)	2491 (17.2%)	3.4
Colorectal	659 (14.0%)	2429 (17.6%)	3.7
Hepatobiliary	622 (13.2%)	1828 (12.9%)	2.9
Upper GI tract	443 (9.4%)	1326 (9.4%)	3.0
Other	1337 (28.5%)	2421 (17.1%)	1.8

## Discussion

This study is the first to rank German general and visceral surgery university hospital departments according to the publication performance of their staff surgeons (chief and consultants). Although Welsch et al. [6] has subjected the publication performance of individual German surgeons to an international comparison, this was not the intent of the present work, which was to benchmark the surgical university departments in Germany.

Regardless of the parameter (publication number, publications per member, generated impact factor or average IF of individual publications from a department), there was considerable difference in department performance. For example, 9 of the 38 departments (1st quartile with the highest number of publications) accounted for approximately 3 times as many publications per member, compared to the last 10 departments (4th quartile) (Fig. 3A). Differences when considering cumulative IF were even more pronounced. The 9 most highly ranked departments generated approximately five times the IF per member, compared to the lowest performing 10 departments (Fig. 4A). Significant discrepancies were not due to the size of the department since there was no relationship between the member number and the number of publications per member (*r* ≈ 0.090, *P* ≈ 0.591) (Fig. 2). The IF per publication also only moderately depended on whether a department had more or fewer members (*r* ≈ 0.451, *P* < 0.005; not shown).

In the present analysis, results were divided into quartiles for benchmarking. Quartiles for each parameter were created separately, meaning that a department in quartile 1 with regard to its publication activity could appear in quartile 2 with respect to IF (and vice versa). Putzer et al. [1] also found that leading departments in terms of publication number did not necessarily lead with the highest average IF per individual publication. Ranking, therefore, depends on the parameter. In the present study, only 8 of 18 departments always found themselves in quartiles 1 or 2 for all four parameters (total publications, cumulative IF, publications per member, and IF per publication). Similarly, only 10 of 20 departments remained in quartiles 3 and 4 in regard to all four parameters. The jump of departments from one quartile to another, depending on the parameter, show that “many publications” cannot be equated with “many publications in journals with high IF.” “Departments with many publications” can also not be equated with “departments with many publications per member.”

Significant differences in publication number have also been revealed for German anesthesiology university hospital departments, whereby it was assumed, but not analyzed, that the publication number depended on the number of members in the department [1]. Schwarzer et al. [7], who examined the publication activity in three medical fields (cardiology, cardiac surgery, general surgery) in Germany from 2011 to 2013, found that cardiologists published most and cardiac surgeons least. The authors concluded that the publication number depended on the number of department members, and found no difference in publication activity among the three disciplines, when referring to publication by each individual member. The finding that more members publish more is to be expected (Fig. 1). More important is the lack of difference in publication activity between the different fields with regard to the number of publications per department member, as shown by Schwarzer et al. [7]. This observation could be confirmed in the present investigation (Fig. 2), whereby a considerable portion of the staff surgeons in both larger and smaller departments was not actively engaged in publication, as far as demonstrated by first or last authorship.

Differing publication activity of academic institutions, such as that shown here for Germany, has also been encountered in other countries. Kahn et al. [8] undertook a comprehensive analysis from January to February 2013 of nearly all academic neurosurgeons and departments within the USA. Data were obtained from 99 departmental websites for a total of 1225 academic neurosurgeons. Departments were ranked by name from 1 to 99 based on the summation of individual faculty h-indices. The publication productivity of 315 neurosurgeons in Great Britain and Ireland was analyzed by Wilkes et al. [9] in January 2014. Academic output was measured by the h-index and stratified in 34 departments.

The academic productivity of 75 spine surgery fellowship-programs from 2011 to 2014 in the USA was analyzed by Schoenfeld et al. [10] The total number of publications per program (2011 to 2014) ranged from 0 to 161, with a median of 12. Only the five most productive programs, ranging in publication activity from 161 to 68 were specifically named. Here the number of fellows in a program (7.0, 0.9–13.2) was significantly associated with the total number of publications.

Geographic differences in both academic promotion and scholarly productivity was noted by Svider et al. [11] among academic otolaryngologists. Practitioners in the West had higher research output than those in other regions. They explained this with a higher emphasis being placed on research productivity among faculty in the West. Hohmann et al. [12] conducted a bibliometric analysis of orthopaedic academic output in the USA. The 15 highest-ranking orthopaedic journals were audited from 2010 to 2014 and then subdivided into anatomic regions and 14 subspecialties. New York led in total publications, greatest activity within subspecialties, and publications per surgeon/population and per median income/capita. Vail, a ski resort in Colorado, led in publications/surgeon and population. The top four cities of New York, Philadelphia, Boston, and Chicago were responsible for 28% of the academic output over the 5-year study period.

In their comparison of cardiology, cardiac surgery, and general surgery, Schwarzer et al. [7] observed that surgeons published in journals with a lower IF than cardiologists. This was attributed to the different number of readers since surgery is comparatively less in demand, thus less cited, and therefore appears in journals with a lower IF. As Table 1 shows, the journals in which most publications appeared were not identical to those with the highest IF. This may be attributed to a targeted German readership reading German-language journals and the fact that IF favors English-language journals. This result was therefore to be expected. Analyzing the focus of publication (Table 3) reveals more about IF distribution. The highest number of publications was devoted to the pancreas, and was about twice that of publications dedicated to the upper gastrointestinal tract. The average IF of individual publications dedicated to pancreas was also significantly higher (4.0) than that for the upper gastrointestinal tract (3.0). Whether this was due to journals with a higher IF preferring to publish pancreas dedicated articles or departments specializing in pancreas surgery publishing more internationally than departments with other focal points, remains open.

The present investigation has limitations, which have been pointed out in the analysis of publication activity in vascular surgery [4]. Only first and last authorships were counted, excluding all other authors placed elsewhere. The publication performance of the current staff surgeons on the target date was assessed, analogous to the studies that were strictly followed for methodological and comparative reasons [2–5]. This means a snapshot was taken on July 1, 2017 and cannot be equated with the publication performance of the individual departments over the 10-year period. For example, if there was a very productive surgeon who retired in 2016 but had been at that University hospital from 2007 to 2016, this individual would not have been counted. This is a major limitation of our study. July 1, 2017 was chosen in order to compare benchmarking in visceral surgery with other fields (vascular surgery and cardiac surgery) in the same time period [4, 5]. Departments with a recent change in management, or departments where consultants had been in non-university positions during the observation period, may be under-represented in publication number if the holder of the position on the target date had published less than the predecessor. Publication activity of residents was not covered in this study, because workplace changes occur frequently within a 10-year period for these physicians. However, they are indirectly included when a chief or consultant of their department was first or last author. This was one reason why not only first but last authorship was also recorded. When for example department directors total 7.7% first authorship (174 out of 2259) and 48.2% last authorship (1476 out of 3104), excluding duplicate accounting for consultant authorship, a significant proportion of publications by other department members not belonging to the staff surgeons, and therefore not captured, are included in the present evaluation. This particularly affects first authorship.

Another controversial factor applicable to the present investigation is that analogous to previous investigations [1–5, 7], publications were rated according to impact factor, which is under discussion as a quality indicator. Since IF is regarded as a quality indicator for a journal, it is also incorrectly regarded as a quality indicator for a single article. This can lead to distortion since one good article that is cited can drive the index up, even if all other articles published in the journal are almost never cited. However, controversy about IF as a quality indicator does not change the fact that the publication performance of individual German university general/visceral surgery departments is very different.

If significant differences in publication performance of the departments examined in the present investigation cannot be explained by size alone, then other reasons must be sought for the disparity. The argument that department members lack time to publish to varying degrees has been negated by Schwarzer et al. [7] on the grounds that the publication number per member was not different in all three of their examined fields. In the present study, the range of publications per member in 38 departments ranged from 21.7 to 2.3 but did not depend on the number of department members. The strongly differing publication achievements of staff surgeons could therefore be explained by differing scientific engagement. This is supported by the high proportion (approximately 20%) of staff surgeons who did not claim first or last authorship over the entire investigated 10-year period. A study by Hinrichs et al. shows that Germany, in terms of its population size [13], exhibits a deficit in English-language publication. These authors examined general surgery publication activity (publication number and IF) and found Germany in sixth place in 2016/2017 (after the USA, Japan, the Netherlands, Great Britain, and France). When publication activity was set in relationship to inhabitant number, Germany was only 12th, far behind countries such as the Netherlands, Sweden, Switzerland, or Denmark.

Manifold circumstances leading to more or less publication activity are conceivable and may vary from university to university. Different organizational and structural forms can influence the emphasis put on scientific activity. An increasing focus on performance-oriented clinical patient care in Germany, with limited research resources and insufficient promotion of science-supporting structures in surgical departments, may also be responsible for sparse scientific activity and may vary greatly from region to region. The increasing shortage of applicants for new surgical positions could also have some effect on scientific activity. Internationally compared, the high percentage of publication-inactive managing team members could explain the moderate position in terms of population that German general/visceral surgery occupies in PubMed-listed publications. Identification of the problems leading to sparse scientific activity should be the goal of future studies and could be approached by national and international comparison not only with other surgical fields, but also with fields outside surgery.

## Conclusion

The publication performance of staff surgeons in academic German general/visceral surgery departments, measured in terms of first and last authorship in PubMed-listed publications, show considerable variation that cannot be explained by the number of department members. Comparative analysis of publication performance provides an objective means of reflecting scientific activity. Further national and international comparative studies should be initiated to identify the causes behind disparity in scientific activity in academic medicine.
